# Simultaneous wall and blood-flow phase-contrast imaging using a single low VENC

**DOI:** 10.1186/1532-429X-14-S1-W49

**Published:** 2012-02-01

**Authors:** Junmin Liu, James A White, Maria Drangova

**Affiliations:** 1 Imaging Research Laboratories, Robarts Research Institute, Schulich School of Medicine & Dentistry, The University of Western Ontario, London, ON, Canada; 2 Division of Cardiology, Department of Medicine, Schulich School of Medicine & Dentistry, The University of Western Ontario, London, ON, Canada; 3 Department of Medical Biophysics, Schulich School of Medicine & Dentistry, The University of Western Ontario, London, ON, Canada

## Summary

The aim of this study is to investigate the feasibility of extracting motion information from a single scan with one low VENC, followed by robust phase unwrapping, for characterizing both intra-ventricular flow and myocardial motion.

## Background

Intra-ventricular blood flow and regional myocardial motion are two key components used in assessing cardiac function. Both can be quantified using phase contrast MRI, but typically require two imaging sequences to be acquired - one with a high velocity encoding value (VENC) and one with a low VENC, selected to optimize velocity sensitivity while avoiding aliasing. In an effort to obtain velocity information from both ventricular blood and myocardial motion in one acquisition, dual-VENC techniques (Buchenberg, ISMRM 2011) have been proposed and evaluated.

## Methods

MR imaging was performed on a 3.0-T whole-body scanner (MR 750, GE Medical Systems). Phase-contrast images with through-plane velocity-encoding were acquired in the short-axis plane with a retrospectively triggered 2D fast cine phase contrast pulse sequence (segmented k-space gradient-echo; TR/TE, 7.3/4.4 ms; flip angle 15 degree, slice thickness 8 mm) with first-order flow compensation in all dimensions to minimize artifacts from flow and motion. Three VENCs - 75, 20 and 10 cm/s - were used and the images acquired with VENC = 75 cm/s were used as a reference. The acquisition time (per VENC) was about 15 seconds, enabling acquisition within a single breath-hold. Thirty images were reconstructed per cardiac cycle.

All images were analyzed off-line using algorithms developed using MATLAB. Phase unwrapping of the velocity data was achieved using an algorithm developed in our lab, which uses an orthogonal recursive approach to remove streaks that result following conventional 2D phase unwrapping.

## Results

**Figure 1:**
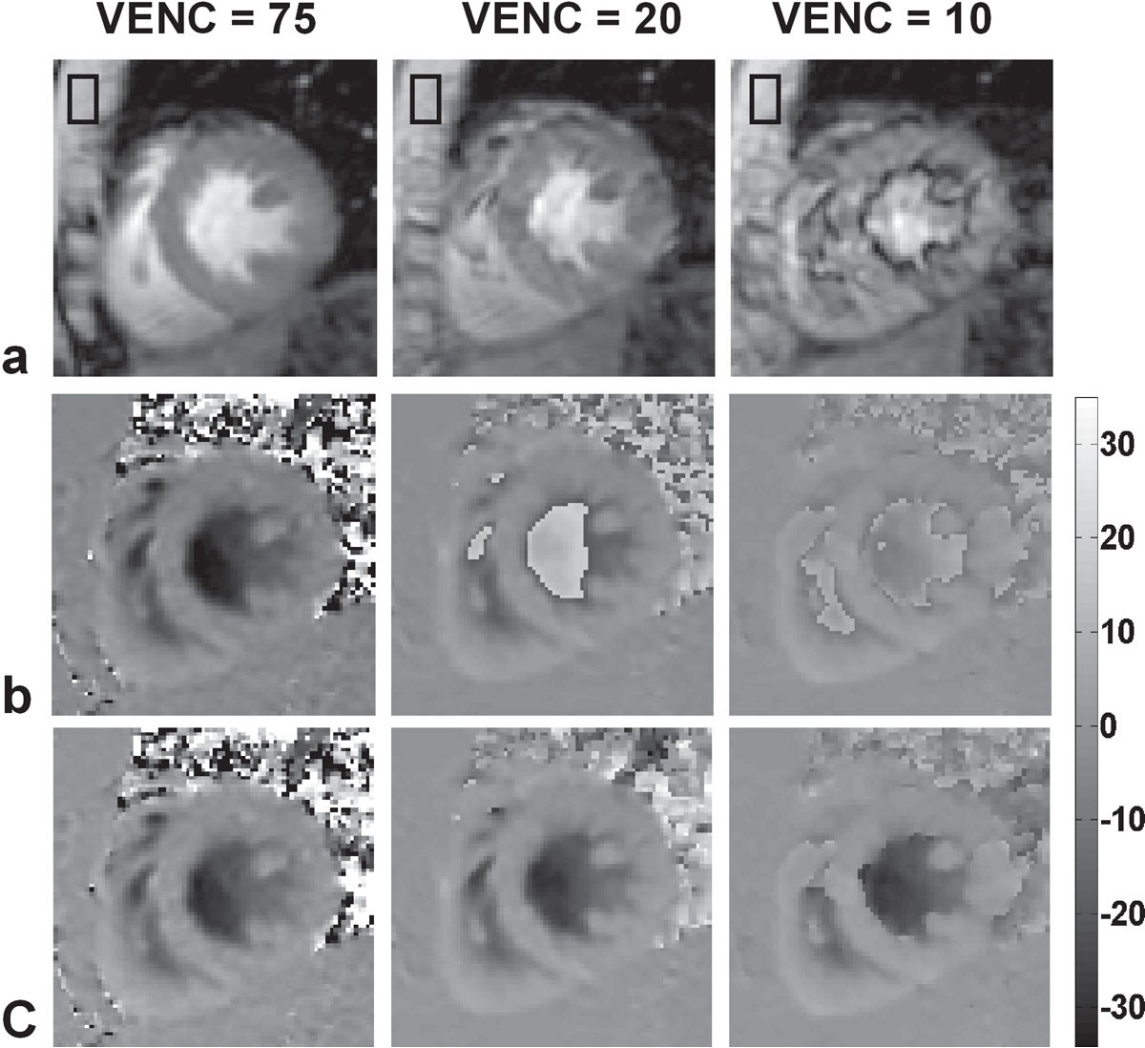
Peak systolic stage: magnitude (a), measured phase (b) and unwrapped phase (c). The phase images have been corrected for background phase (based on the mean phase value within the boxes drawn) and are scaled in cm/s according to the scale on the right.

**Figure 2:**
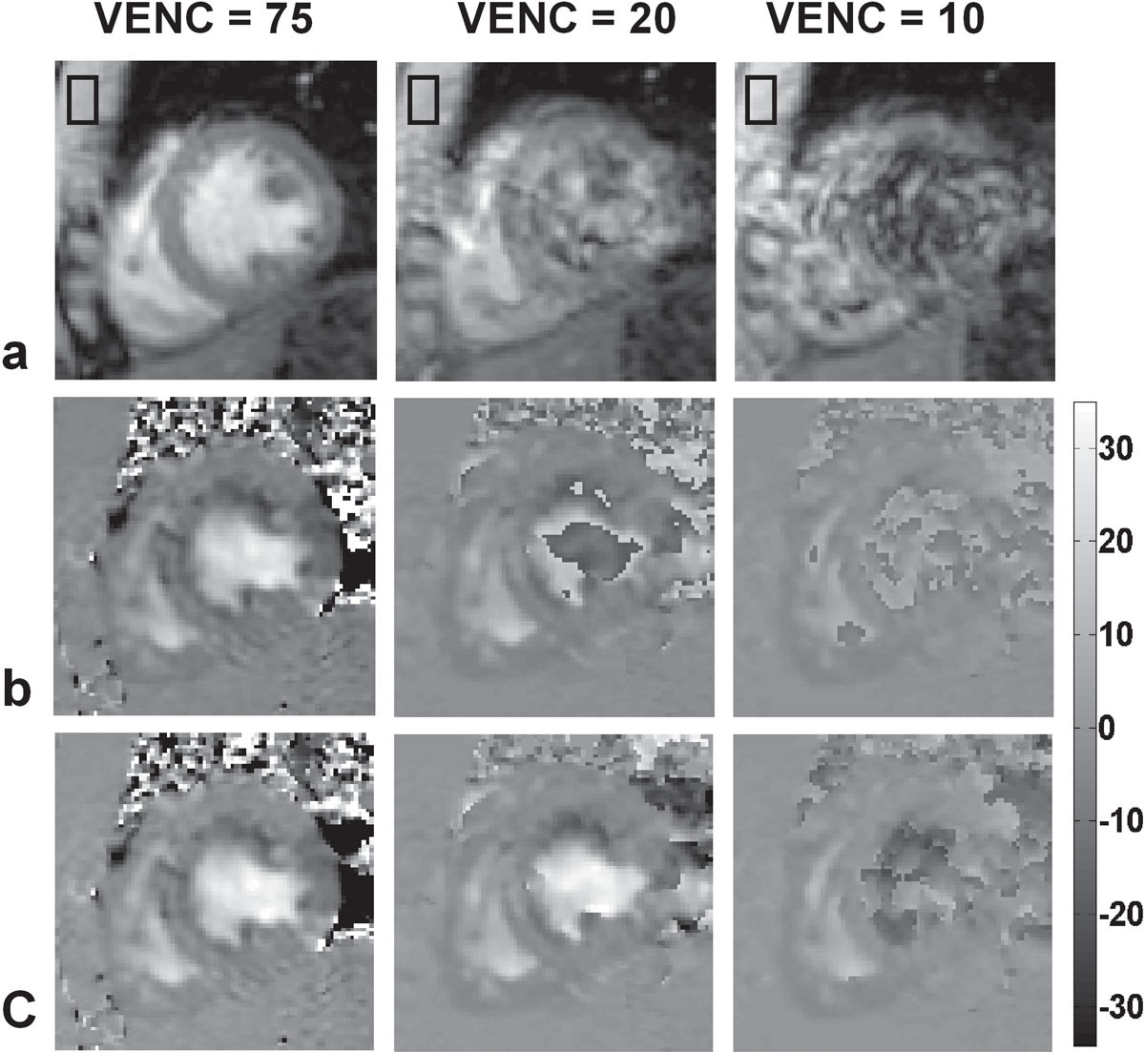
Early-filling stage: magnitude (a), measured phase (b) and unwrapped phase (c). The phase images have been corrected for background phase (based on the mean phase value within the boxes drawn) and are scaled in cm/s according to the scale on the right.

Mid-ventricular phase-contrast images corresponding to peak systole and early filling are shown in Figures 1 and 2, respectively. Severe phase aliasing is seen within the LV blood pool when VENCs of 20 and 10 cm/s were used (Figures 1b and 2b). Our technique successfully unwrapped the images acquired with VENC = 20 cm/s but failed with VENC = 10 cm/s for the early-filling stage. The flow velocity from phase images acquired using VENC = 20 cm/s are quantitatively similar to the reference phase images but with lower noise in heart wall compared to that of VENC = 75 cm/s (submit to SCMR 2012).

## Conclusions

The results suggest that single low-VENC (>= 20 cm/s) acquisitions can be successfully used to measure intra-ventricular flow and wall motion simultaneously.

